# Canonical Babbling: A Marker for Earlier Identification of Late Detected Developmental Disorders?

**DOI:** 10.1007/s40474-019-00166-w

**Published:** 2019-05-30

**Authors:** Sigrun Lang, Katrin D. Bartl-Pokorny, Florian B. Pokorny, Dunia Garrido, Nivedita Mani, Annette V. Fox-Boyer, Dajie Zhang, Peter B. Marschik

**Affiliations:** 1 iDN – interdisciplinary Developmental Neuroscience, Division of Phoniatrics, Medical University of Graz, Auenbruggerplatz 26, 8036 Graz, Austria; 2 Machine Intelligence & Signal Processing group, Chair of Human-Machine Communication, Technical University of Munich, Munich, Germany; 3 Mind, Brain, and Behavior Research Center, University of Granada, Granada, Spain; 4 Psychology of Language Department, Georg-August University Göttingen, Göttingen, Germany; 5 Leibniz-ScienceCampus Primate Cognition, Göttingen, Germany; 6 Department of Human Communication Sciences, Sheffield University, Sheffield, Great Britain; 7 Child and Adolescent Psychiatry and Psychotherapy, University Medical Center Göttingen, Göttingen, Germany; 8 Center of Neurodevelopmental Disorders (KIND), Center for Psychiatry Research, Department of Women’s and Children’s Health, Karolinska Institutet, Stockholm, Sweden

**Keywords:** Autism spectrum disorder, Canonical babbling, Late detected developmental disorders, Early detection, Fragile X syndrome, Rett syndrome

## Abstract

**Purpose of Review:**

To summarize findings about the emergence and characteristics of canonical babbling in children with late detected developmental disorders (LDDDs), such as autism spectrum disorder, Rett syndrome, and fragile X syndrome. In particular, we ask whether infants’ vocal development in the first year of life contains any markers that may contribute to earlier detection of these disorders.

**Recent Findings:**

Only a handful studies have investigated canonical babbling in infants with LDDDs. With divergent research paradigms and definitions applied, findings on the onset and characteristics of canonical babbling are inconsistent and difficult to compare. Infants with LDDDs showed reduced likelihood to produce canonical babbling vocalizations. If achieved, this milestone was more likely to be reached beyond the critical time window of 5–10 months.

**Summary:**

Canonical babbling appears promising as a potential marker for early detection of infants at risk for developmental disorders. In-depth studies on babbling characteristics in LDDDs are warranted.

## Introduction

Speech-language acquisition typically follows a defined domain-specific pathway cascading from one milestone to the next one, increasing in complexity, accuracy, and stability. During the first year of life, infants’ receptive and productive capacities develop from being universal to language-specific [1–4]. Early vocal development follows a sequence to build up our complex human speech capacity [5–11] as well as language and socio-communicative functions (e.g., lexical acquisition, phonological awareness, literacy skills [12–15]). Irrespective of differences in underlying theoretical linguistic frameworks or explanatory models, there is agreement that it all starts with the first cry, followed by the production of vegetative and quasi resonant sounds before the first cooing sounds occur at around 3 months of age. Infants typically move on to produce fully resonant sounds, raspberries, and marginal syllables, and then canonical syllables which form the basis of first spoken words that usually appear around the first birthday. In the second year of life, complexity of vocal production increases [9–11, 16], and the initially rather slow growth of the expressive vocabulary rapidly increases to a vocabulary spurt or similar fast-mapping related vocabulary growth patterns and first word combinations [17–20]. The continuously increasing proficiency of the speech-language and communicative capacity across all linguistic levels—phonetics, phonology, semantics, morphology, syntax, and pragmatics—leads up to evolving fairly competent speakers at school age.

The most prominent and among the most extensively studied speech-language milestones before the first spoken words is the emergence of canonical syllables [3, 7, 21, 22•]. In contrast to precanonical vocalizations (e.g., quasi-resonant sounds, cooing sounds, fully-resonant sounds or raspberries), canonical syllables consist of a consonant- and a vowel-like part. Additionally, canonical syllables are target-language-like and thus differ from marginal syllables, which are produced with slow or erratic formant transitions [7, 11, 23]. Canonical babbling is characterized by syllables with at least one vowel-like element and one supraglottal consonant-like element with a rapid, adult-like formant transition between consonant and vowel (phonetical representation: e.g., [ba], [di], [ata], [nunu], [dada]; [24, 25]). The rapid transition between consonants and vowels is a defining feature of the difference between precanonical and canonical syllable productions. Infants gradually develop the oral-motor skills necessary to produce adult-like consonant-vowel-syllables, which, in turn, are the prerequisite to uttering conventional words [7, 26]. Another characteristic of vocalizations in the babbling period is multisyllabicity [8, 21]. Notably, some authors have differentiated between reduplicated (e.g., [dada], [mamamam]) and variegated babbling (e.g., [abababedada]; [5, 6]) while others have subsumed both under the term *canonical babbling* [11]. Given this inconsistence in terminology, in this paper, we use the term *canonical babbling* to embrace single consonant-vowel-syllables as well as disyllables and strings of syllables, either reduplicated or variegated.

In addition to these divergent definitions, there are also inconsistencies in the parameters chosen to define the onset of canonical babbling. While some researchers used the definition of a consonant-vowel-syllable with rapid transition between the consonant and the vowel and defined a canonical babbling ratio (CBR) of 0.20 (CBR^utt^ = number of canonical syllables/total number of utterances; [27]) or 0.15 (CBR^syl^ = number of canonical syllables/total number of syllables; [28] and CBR^UTTER^ = number of utterances containing canonical syllables/total number of utterances; [29]) as the onset, others considered canonical babbling as established if at least one or two canonical syllables or strings of canonical syllables occur in a speech sample of 10–45 min length [21, 30, 31]. Additionally, Schauwers et al. suggested a *stability parameter* required for the establishment of the canonical babbling phase defined through the presence of at least two vocalizations with multiple articulatory movements in one respiration cycle over three consecutive months [31], labeled *MULTI* by Molemans et al. [32]. Also, studies differ, for example, in whether to include [11, 27, 28] or exclude the approximants [ʋ] and [j] as true consonants in canonical syllables [33, 34]. Based on the exclusion of these phonetic realizations, a true canonical babbling ratio (TCBR) of 0.20 (TCBR^utt^ = number of true canonical syllables/total number of utterances; [32]) and 0.15 (TCBR^syl^ = number of true canonical syllables/total number of syllables; [34]) was proposed as onset measurement.

Nevertheless, based on parental reports and video-analysis at home or in laboratory settings, canonical babbling onset (CBO) in typically developing (TD) infants has been observed between 5 and 10 months of age [6, 35]. Moreover, this age range for CBO appears to be relatively consistent across languages, as reported for English, Dutch, Swedish, Spanish, and German, to name but a few [9–11, 32, 36]. Data from Mandarin- or Cantonese-learning infants revealed a similar age range of CBO [37], with slightly higher CBR^syl^-values as compared to English-learning infants [38••]. Thus, like many other developmental phenomena, age of CBO may reveal considerable individual variation, while nevertheless pointing to a critical window before 10 months of age. The conditions that lead to late babbling may set off a cascade, negatively affecting a variety of additional linguistic capabilities down the road. The core problem that causes late babbling could generalize to other aspects of language and literacy development. Thus, the absence of canonical babbling and persistence of precanonical vocalizations as the predominant vocalization pattern beyond 10–12 months of age may indicate atypical vocal development and precede later speech and language deviations [25, 39]. Indeed, Oller et al. reported that infants who did not produce canonical syllables at 10–12 months also had a reduced expressive vocabulary at 18, 24, and 30 months of age [24]. Eilers and Oller found that infants with hearing impairment failed to produce canonical syllables until 11 months of age, while all TD infants reached this milestone by 10 months [35]. Delayed or deviant babbling in infants with hearing impairment has also been reported in other studies [40–44]. A later onset of canonical babbling has also been observed in other developmental disorders such as in infants with Williams syndrome [45, 46], Cri du Chat syndrome [47], and childhood apraxia of speech ([48]). Interestingly, in infants with Down syndrome, evidence exists both for and against a delay in CBO [49–52], suggesting that achieving the canonical babbling milestone within the critical time window does not necessarily lead to typical speech and language outcome.

While Down syndrome can be identified prior to or at birth, and early developmental profiles can thereafter be easily documented, great challenges arise when dealing with developmental disorders commonly diagnosed beyond toddlerhood. Prodromal development in babbling has received much less attention in late detected developmental disorders (LDDDs), such as autism spectrum disorder (ASD), Rett syndrome (RTT), and fragile X syndrome (FXS). The methodological difficulties of *looking back* on the pre-diagnostic development are further exacerbated by the fact that many LDDDs are also rare diseases, leaving few participants available for investigation [53–55, 56••]. Nonetheless, as delayed or atypical babbling is often associated with later verbal deficits [24, 25, 33, 57], infants with LDDDs and associated speech-language and socio-communicative deficits may well exhibit peculiarities in vocal development early in life. Understanding typical and atypical characteristics and trajectories of vocal development in the first year of life may in turn contribute to detecting these LDDDs at an earlier time. In a recent paper, Roche et al. reviewed studies on early vocalizations of infants with such LDDDs [58••]. While not exclusively focused on babbling, the authors suggested a reduced likelihood that infants with LDDDs would reach the canonical babbling stage. In this article, we chose to focus on infants later diagnosed with ASD, RTT, and FXS—conditions sharing deviances in speech-language and socio-communicative development. We aim to provide an overview of studies investigating the onset and characteristics of canonical babbling in children with these LDDDs. We highlight the importance of investigating such early markers which may prove to be valuable components of early screening and diagnosis of infants at risk for developmental disorders.

### Autism Spectrum Disorder (ASD)

Autism spectrum disorder is a developmental disorder characterized by persistent deficits in social interaction and communication, and the presence of restrictive and repetitive behaviors [59]. In the USA, ASD prevalence was reported to be 16.8 in 1000 children aged 8 years [60]. The specific causes driving the atypical neurodevelopment of ASD remain poorly understood. A number of candidate genes (e.g., *NRXN*, *PTCHD1*, *SHANK*; [61]) as well as environmental factors (e.g., maternal infections or medication during pregnancy, advanced parental age, environmental pollutants; [62, 63] are assumed to play a role in the etiology of ASD. A recent parent survey found the mean age of ASD diagnosis remains rather late in the toddlerhood [64].

Studies on the emergence and production of canonical babbling in infants later diagnosed with ASD report inconsistent findings, in part, due to the different research methods applied and definitions used. For example, Werner et al. retrospectively analyzed home videos and found no different frequencies of canonical syllables per minute between the ASD and the control group at 8–10 months, yet found a group difference at 12 months in frequencies of complex babbling [65]. Chericoni et al., also using retrospective video analysis (RVA), studied infants from 0 to 18 months. They categorized infants’ vocal behaviors into four groups: (1) vocalizations (vowels or non-reduplicated consonants and vowels), (2) long reduplicated babbling (three or more units), (3) two-syllable babbling, and (4) first words [66]. In contrast to the previous findings by Werner et al., while between 6 and 12 months, infants with ASD and TD infants did not differ in rate (counts per minute) of producing canonical syllables (i.e., group (2) and (3)), infants with ASD produced less precanonical vocalizations (i.e., group (1)) than the controls. In yet another RVA study, however, Patten et al. calculated the CBRs of infants aged 9–12 and 15–18 months [67]. They found significantly lower CBRs and lower volubility for the infants with ASD compared to TD controls. In a prospective study, Paul et al. examined vocalizations in infants with an older sibling diagnosed with ASD (i.e., the high-risk group) [68]. They investigated the percentage of canonical syllables (number of true consonant-vowel-syllables/total number of speech-like vocalizations) and found a significantly lower CBR at 9 months in the high-risk group than in the low-risk controls. At the age of 12 months, however, this difference disappeared. In another prospective study, Pokorny et al. reported that out of ten infants with ASD aged 10 months, four produced vocalizations more complex than single canonical syllables, which was comparable to typical controls [69•]. Neither did the authors find a significant difference in volubility of overall vocalizations between the two groups. LeBarton and Iverson videotaped infants at high risk for ASD with their primary caregivers monthly between 5 and 14 months of age [70]. They found that 33 of 37 infants at high risk for ASD reached the canonical babbling milestone (i.e., first observation of reduplicative babbling). Mean onset age of canonical babbling was 7.67 months (SD 1.76, range 5–12). Iverson and Wozniak, on the other hand, found a mean onset age of canonical babbling (i.e., regular use of reduplicative babbling) between 5 and 18 months for high-risk infants and between 5 and 9 months for low-risk controls, with a significantly higher portion of the high-risk infants showing a CBO at the later end of the age range [71]. In sum, while infants with ASD often achieve the canonical babbling milestone, the onset time, rate, and other characteristics of their babbling development frequently reveal deviations.

### Rett Syndrome (RTT)

Rett syndrome is an X-linked genetic disorder with a prevalence of approximately 1 in 10,000 female births [72]. The main genetic cause of RTT are de novo mutations in the Methyl-CpG-binding protein 2 gene (*MECP2*; [73]). For a long time, RTT was characterized by a rather inconspicuous early development followed by the partial or complete loss of already acquired purposeful hand skills and spoken language. However, as *MECP2* mutations potentially affect prenatal and postnatal brain development [74, 75], a perspective change was called for to acknowledge an earlier onset of symptoms opposing the typical early development assumption [76–82]. Based on the consensus criteria released in 2010, RTT is divided into typical/classic and atypical RTT/variants [81]. A relatively mild RTT phenotypical appearance associated with comparably better speech-language capacities (i.e., use of a number of single words or even word combinations/phrases) than seen in typical RTT, relatively better functional hand use, and milder intellectual disability is the preserved speech variant (PSV; [81, 83–85]). Still, individuals with PSV follow an atypical speech-language development including the loss of acquired functions [79, 82, 85, 86]. The current mean age of diagnosis is 2.7 years for typical RTT and 3.8 years for atypical RTT [87].

Systematic research on early vocalizations in general and canonical babbling in particular in individuals with RTT is scarce. When parents were asked to recall their child’s earlier development, the majority were convinced that the child had produced canonical babbling or first words prior to regression [88, 89]. More objective analyses utilizing RVA to assess canonical babbling, to the best of our knowledge, has been described in only three studies [79, 90, 91]. Overall, results revealed that girls with RTT show poor verbal development and they are likely to achieve the canonical syllables milestone with delay, if at all. Out of six infants with PSV (four Italian- and two German-speaking) investigated by Marschik et al., five failed to produce well-formed syllables/canonical babbling-like vocalizations until 12 months of age [90], two did not show CB until the second birthday, and only one girl of the six infants in this study produced canonical babbling at an age of 7 months, which falls within the typical age range. This girl achieved extraordinarily high language proficiency very rarely seen in RTT [86, 92]. Einspieler et al. studied a pair of monozygotic twins with typical RTT (Portuguese-speaking) [91]. They did not observe canonical babbling until the children were 21 months. A third study revealed that six out of 15 individuals with RTT did not produce any canonical babbling during their first 24 months of life [79]. While half of the infants with typical RTT reached this milestone (5/10 cases), the proportion was significantly higher in the comparably milder PSV (4/5 cases). It is important to note that the authors not only reported absence or delay of achieving expressive verbal milestones but also qualitative differences or typical vocalizations interspersed with atypical phonetic realizations (e.g., vocalizations on inspiratory airstream, high pitched crying; [79, 90, 91, 93, 94]. As only retrospective studies on vocal behaviors could be conducted thus far, the age of CBO in RTT is yet to be determined, since complete sampling is not obtainable. Also, CBRs or volubility counts have not been reported for RTT to date. In sum, a proportion of infants with RTT do reach the canonical babbling stage, yet the majority of them experiences considerable deviation and delay.

### Fragile X Syndrome (FXS)

Fragile X syndrome is the leading cause of inherited intellectual disability and the most common monogenic cause of ASD [95–98]. FXS was estimated to occur in 1 of 5000 males and in 1 of 4000 to 8000 females [99]. FXS results from the excessive repeat of a trinucleotide CGG at the Xq 27.3 site on the Fragile X Mental Retardation-1 gene (*FMR1*; [98, 100]). The mean age of FXS diagnosis remains at about 3 years [101].

To the best of our knowledge, only two RVA studies have investigated babbling in infants with FXS [102•, 103•]. In one study, two out of seven infants (5 monolingual German-, 2 bilingual German-Spanish-speaking) were found to produce canonical babbling between 9 and 12 months of age [103]. In the other study, none of the ten infants (English-speaking households) reached the milestone of canonical babbling (defined as reaching or exceeding a CBR^syl^ of .15; [102•]). In this study, however, eight of the 14 typically developing controls aged 9–12 months also failed to reach this milestone.

## Discussion

With this paper, we aimed to provide an overview of studies on canonical babbling in infants with late diagnosed developmental disorders. We exemplarily chose ASD, RTT, and FXS, as all these LDDDs are speech- and language-related disorders. Leaving aside issues of methodological differences and definitions used for the relevant measures, the few existent studies reviewed here suggest that infants with ASD, RTT, or FXS are likely to show deviant patterns of babbling development compared to TD infants.

To understand the prediagnostic development of LDDDs, many researchers have relied on RVA for good reasons [54]. It is only in the last decade that prospective studies have boomed rapidly, providing us novel opportunities to investigate speech-language development from early on (i.e., high-risk ASD studies). As has been discussed elsewhere, RVA inherently suffers from sampling limits [54, 104, 105], making determination of the onset and the presence/absence of a specific behavior (e.g., canonical babbling) elusive. While prospective studies are certainly the method of choice to study high-risk cohorts, less prevalent disorders or disorders with unknown etiology or without hereditary pathways will—at least for now—still be studied applying retrospective approaches.

Findings of the scarce babbling studies on ASD, RTT, and FXS were, in the face of all methodological difficulties, in line with previous studies investigating infants with or at risk for developmental disorders [24, 25, 33, 57]. The production of canonical syllables is an essential and necessary step in a child’s pathway to the spoken world. A delayed CBO or deviant canonical babbling pattern appeared to be a precursor of further atypical speech-language development. Notably, in all our targeted groups (i.e., ASD, RTT, and FXS), there were infants who did achieve the babbling milestones within the typical time window. This suggests that although absent or delayed canonical babbling proved alarming, the presence of babbling before 10 months of age does not predict later intact speech and language skills. It remains possible that adverse characteristics of this early vocal behavior might hide behind a timely CBO, and qualitative distinctions in babbling may associate with the severity of later language deficits. This deserves further in-depth research with more advanced methodology.

Deviances or delay in canonical babbling is highly specific but not sensitive enough to refer to atypical language development. A deficit in early development, however unfortunately, appears seldom alone. A deleterious sign might often precede or coexist with the others. As distressful as it can be for the individual of concern, if one sign appears subtle or elusive, multiple signs within and across domains might flag atypical development of an infant to caregivers and practitioners, which solicits earlier detection and intervention.

## Conclusions

The increasing evidence of “babbling-alterations” in infants with LDDDs indicated that canonical babbling, as one of several milestones bootstrapping speech-language development, may prove to be another valuable marker for earlier detection of these disorders. Beyond the investigation of the presence or absence, and the onset age of canonical babbling in infants with LDDDs, it might be of clinical importance to examine the potential qualitative differences of infant babbling and its relation to later language outcomes. Upcoming research may benefit from capturing acoustic representations of babbling and other infant vocal behaviors in LDDDs [56••, 69•, 94, 106–109]. Combining concurrent audio signal processing methods with machine learning technology [110] is a promising approach for objective automated recognition of peculiarities and deviances in atypical development. Future systematic approaches netting canonical babbling and other markers in early development may facilitate better understanding and timely identification of different pathways leading to specific disorders.
